# Experimental models for intestinal host-microbe interactions

**DOI:** 10.1038/s44321-026-00450-4

**Published:** 2026-05-21

**Authors:** Yuqi Li, Naschla Gasaly, Paul de Vos

**Affiliations:** Centre for Healthy Eating & Food Innovation (HeFi), Sustainable Foods and Health, Faculty of Science and Engineering, Maastricht university, Venlo, The Netherlands

**Keywords:** Methods & Resources, Microbiology, Virology & Host Pathogen Interaction

## Abstract

The intestinal barrier is a dynamic interface integrating epithelial integrity, mucus architecture, and immune signaling to maintain host homeostasis. While microbial metabolites such as short-chain fatty acids, secondary bile acids, and tryptophan derivatives regulate this system, translating these insights to human physiology is hindered by experimental models that fail to capture the intestine’s full biological complexity. In this review, we conceptualize intestinal barrier failure as a sequential process comprising junctional remodeling, mucus depletion, and immune-driven permeability. We critically evaluate current in vitro and ex vivo models, highlighting how metabolic biases and reductionist designs in common platforms limit their predictive value. We argue that the primary bottleneck is not a lack of model diversity, but the absence of integrative, human-relevant strategies. Consequently, we propose a stepwise framework linking dynamic microbial fermentation to mechanistic epithelial systems and ex vivo human tissues. This approach moves beyond descriptive modeling toward functionally predictive platforms that align microbial metabolism with host responses across biological scales, ultimately informing clinical translation, precision nutrition, and therapeutic development.

## Abstract (web summary)

In this review, P. de Vos and colleagues discuss how intestinal barrier failure arises, critically assess limitations of current experimental models, and propose an integrative, human-relevant framework linking microbial metabolism to host responses to enable more predictive and translational research.

## Graphical abstract

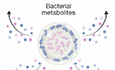

## Introduction

The intestinal barrier functions as a sophisticated, multi-layered interface that maintains the homeostasis between the host’s internal milieu and the diverse microbial ecosystem of the lumen (Mowat and Agace, 2014). It is not only a static physical barrier of epithelial cells, but also represents a functional community comprising the biochemical mucus layer, the specialized epithelial lining, and the underlying mucosal immune compartment. Disruption of this architecture has been confirmed to cause various pathologies, ranging from inflammatory bowel disease (IBD) and metabolic syndromes to neurodegenerative disorders (Neurath et al, 2025). In this dynamic system, microbial metabolites, such as short-chain fatty acids (SCFAs), secondary bile acids (SBAs), and tryptophan metabolites, serve as critical signaling molecules that modulate intestinal epithelial turnover, junction integrity, and immune tolerance (Ornelas et al, 2022).

Despite the growth of data on microbial-host interactions, applying these findings to humans remains challenging because the currently available experimental models often do not match human biology. In addition, current studies often focus on the integrity and disruption of the barrier, failing to capture the temporal sequence through barrier failure. Specifically, an early phase of junctional remodeling and cytoskeletal tension changes, followed by an intermediate phase of mucus layer thinning, and culminating in late-phase immune activation and cytokine-driven permeability amplification. Understanding the strategic selection and optimization of experimental models is therefore central to decoding these temporal dynamics of intestinal host-microbe interactions.

The rationale for focusing on in vitro and ex vivo systems stems from the inherent limitations of current in vivo approaches. Directly studying these processes in the human gut is limited by limited tissue accessibility, ethical restrictions, and high variability between individual donors. While animal models are frequently used, they present substantial interspecies differences in microbiota composition, mucosal physiology, and immune signaling. Consequently, results obtained in rodents or gnotobiotic systems limit the translational value of in vivo findings.

In this review, we discuss human-based in vitro and ex vivo platforms to critically evaluate the technical frontiers of reconstructing human-like intestinal environments. We propose a stepwise framework that organizes these models based on which stage of barrier breakdown they can measure. By tracking specific biological markers such as oxidative stress, protein folding errors (UPR), and the layout of cytokine signaling, we evaluate how well models like organoids, dynamic fermentation models, anaerobic co-culture models, gut-on-a-chip, and human tissue-based models can predict the clinical outcomes. We aim to provide a clear guide for choosing the appropriate model to study the intestinal host-microbe interactions.

## Mechanistic interpretation of barrier function and signal transduction

### The temporal progression of intestinal barrier failure

The intestinal barrier is not a static gatekeeper but a living assembly that undergoes a complex lifecycle of barrier failure in response to microbial dysbiosis. The barrier failure is a sequential process rather than a simple binary event of barrier integrity and disruption, which begins with three phases: (1) Phase I: junctional remodeling, characterized by the spatial reorganization of tight junction (TJ) proteins, such as occludin and claudins, and changes in cytoskeletal tension (Ornelas et al, 2022). These early events often occur before a significant decrease in transepithelial electrical resistance (TEER), marking it a critical mechanistic turning point. (2) Phase II: mucus depletion involves the biochemical erosion of the glycocalyx and mucus layers. This loss of physical separation allows pathobionts to transition from the lumen to direct epithelial contact. (3) Phase III: immune-driven permeability represents the terminal collapse of the barrier, where microbial translocation triggers the release of pro-inflammatory cytokines such as TNF-α and IL-6. These signals drive epithelial apoptosis and irreversible permeability. Recognizing this sequence allows for a more precise mapping of how specific metabolites intervene at different depths of the barrier architecture.

### Receptors mapping and metabolite-driven barrier regulation

The transition between the phases of barrier failure is regulated by microbial metabolites through specific receptors. The outcome of these signaling pathways is fundamentally dictated by the precise cellular distribution of receptors across the intestinal epithelial and immune cell compartments. Therefore, providing a structured mapping across intestinal compartments is essential for defining the physiological relevance of the experimental model (Table 1).

**Table 1:** Structured mapping of key metabolite receptors across intestinal compartments.

Receptor type	Key receptor	Primary cell type	Major metabolites	Barrier function (phase relation)	References
Nuclear receptor	AhR	IECs, macrophages, Tregs, Neutrophils	Indoles (IPA/IAld)	Stimulates mucus and AMPs secretion (Phase II) and promotes IL-22/IL-17 production (Phase III).	Sun et al, 2020; Vazquez-Gomez et al, 2023
	PXR	IECs	SBAs (LCA); Indoles (IPA/IAld)	Indoles promote TJ protein assembly (Phase I) and suppress intestinal inflammation (Phase III).	Fu et al, 2023; Liu et al, 2025
GPCR	TGR5 (GPBAR1)	Enteroendocrine cells, IECs, DCs, macrophages	SBAs (LCA/DCA)	Promotes GLP-1 secretion and suppresses inflammasome activity (Phase III) under physiological conditions.	Hu et al, 2021; Tian et al, 2022
	GPR41/43	IECs, DCs, Macrophages, B cells, ILC3s, Tregs	SCFAs (Acetate/Propionate/Butyrate)	Modulates NLRP3 inflammasome and induces expression of IL-18, AMPs and TJ proteins (Phase I, III).	Mann et al, 2024
	GPR109A	IECs, Macrophages, DCs	SCFAs (Butyrate)	Enhances TJ proteins assembly and reduces pro-inflammatory cytokine levels (Phase I, III).	Mann et al, 2024; Wang et al, 2026
Pattern recognition receptors (PRR)	TLR4	IECs, Macrophages	LPS	Induced the inflammatory storm via epithelial NF-κB activation (Phase III)	Hausmann et al, 2021
	TLR5	IECs, DCs, Monocytes	Flagellin	Reduces pro-inflammatory cytokine levels (Phase III) and enhances the physical barrier (Phase II)	Feng et al, 2023
	TLR2	IECs, Regulatory B cells	Peptidoglycan	Attenuates intestinal inflammation via IL-10 production	Lee et al, 2026; Li et al, 2022

**Figure 1:**
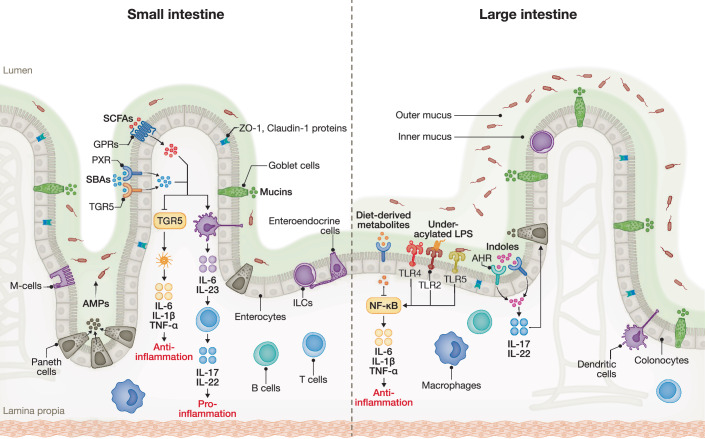
Regional specialization of the intestinal immune barrier in the small and large intestine. The small intestine and colon exhibit distinct physiological and immunological characteristics that together maintain mucosal homeostasis. In the small intestine, Paneth cells secrete AMPs, and the thinner mucus layer permits limited microbial contact. Microbial metabolites such as SCFAs and SBAs modulate epithelial and immune signaling through GPRs, PXR, and TGR5, regulating NF-κB activity and cytokine production (IL-6, IL-1β, IL-23), thereby balancing anti-inflammatory and pro-inflammatory responses (Th17 and IL-22 pathways). In contrast, the large intestine is characterized by a thick, two-layered mucus structure, where bacteria colonize the outer mucus layer. Colonic epithelial cells detect microbial-associated molecular patterns (MAMPs), such as hexa-acylated LPS, through TLR4 signaling, while indole-derived metabolites act via AhR and PXR to suppress excessive inflammation by promoting AMPs secretion. Together, these regional differences reflect the small intestine’s role in active immune sensing and antimicrobial defense, versus the colon’s function in tolerance maintenance and metabolite-driven immune modulation.

Microbial metabolites directly regulate the physical and biochemical integrity of the intestinal barrier through these molecular pathways. SCFAs, particularly butyrate, reinforce the epithelial scaffold by upregulating the transcription and localization of TJ proteins such as ZO-1 and Claudin-1. These effects are primarily mediated via ligands of G protein-coupled receptors (GPR), such as GPR41, GPR43, and GPR109A, which stabilize the Phase I (Perez-Reytor et al, 2021). Similarly, tryptophan-derived indoles, including indole-3-propionic acid (IPA) and indole-3-aldehyde (IAld), activate the aryl hydrocarbon receptor (AhR) and pregnane X receptor (PXR), which not only promote epithelial resilience but also stimulate the secretion of antimicrobial peptides (AMPs) into the mucus layer, thereby enhancing the Phase II defense (Hou et al, 2018; Palrasu et al, 2025). Notably, the signaling effects of SBAs exhibit a distinct “biphasic” dose-dependent effect that underscores the interdependence of the intestinal barrier. At lower physiological concentrations, SBAs support epithelial homeostasis primarily via the activation of the G protein-coupled receptor TGR5 (also known as GPBAR1) (Hu et al, 2021). At higher levels, SBAs can induce membrane and mitochondrial stress, directly increasing paracellular permeability and triggering inflammatory responses (Fiorucci et al, 2022). Furthermore, disrupted mucus homeostasis can markedly attenuate the receptor-mediated benefits of SBAs in Phase II, allowing these metabolites to exert rapid damage to the underlying intestinal epithelial cells (Zhang et al, 2023). This highlights the necessity of using integrative models that capture both the mucus layer and receptor signaling to avoid misleading conclusions regarding metabolite toxicity or protection. While these receptor-mediated pathways provide the initial molecular response to microbial metabolites, their ultimate impact on the intestinal barrier is determined by their convergence onto a shared intracellular regulatory system (Fig. 1).

### Conserved mechanistic axes from intracellular stress to tissue-level signaling

Although the physical signs of barrier failure are phase-dependent, the underlying biological signals can be tracked across all experimental models through conserved mechanistic axes. This continuity ensures that readout remains similar whether it is derived from a simple fermentation system or a complex tissue slice. Importantly, these axes are not isolated but a hierarchical signaling network that translates initial microbial stimuli into terminal biological outcomes.

The first axis involves proteostasis and endoplasmic reticulum (ER) stress. This system acts as a primary intracellular sensor of external pressure, operating through a coordinated signal transduction system known as the UPR (Di Mattia et al, 2025). Disruptions in protein folding capacity often serve as an early signal of epithelial distress, before structural damage (Salamone et al, 2025). By monitoring the expression of key molecular markers such as GRP78 and activating transcription factor 4 (ATF4), researchers can capture early signals when microbial metabolites first interact with the epithelium, bridging metabolite stimulus and cellular responses. Then, this internal stress intersects with the second axis, the cellular redox state, which reflects the metabolic condition of the tissue (Lin et al, 2022). A model’s relevance is defined by its ability to maintain the oxidative phosphorylation-driven environment of a healthy colon instead of the glycolytic bias common in many laboratory-adapted cell lines.

The transition from these intracellular signals to systemic immune activation is mediated by the convergence of diverse receptor-level inputs onto the intracellular regulatory hub. Metabolite receptors such as AhR, PXR, and GPR intersect at the core transcriptional level, most notably the NF-κB pathways (Feng et al, 2025; Li et al, 2025; Wang et al, 2024). For example, while GPR-mediated SCFAs typically promote barrier integrity, their efficacy is regulated by the activation of the AhR/NRF2 pathway, which recalibrates the epithelial redox state (Rzeczycki et al, 2025). Notably, the activation of this hub is also regulated by the structural determinants of microbial ligands like microbial-associated molecular patterns (MAMPs), including LPS, peptidoglycan fragments, and flagellin. Unlike the hexa-acylated LPS from pathogens that trigger robust NF-κB-mediated inflammation (Manivannan et al, 2025), the under-acylated LPS fail to induce TLR4/myeloid differentiation factor-2 (MD2) dimerization, which decouples microbial sensing from inflammatory signaling, shifting it toward immune tolerance (Zamyatina and Heine, 2020).

The final axis is the topology of cytokine networks, which introduces a holistic readout of the mucosal environment. This system focuses on the interactions between pro-inflammatory, anti-inflammatory, and tissue-repair signals. For instance, the balance between pro-inflammatory cytokines such as TNF-α and IL-1β and anti-inflammatory mediators, including IL-10 and TGF-β (Cristofori et al, 2021; Fernandez-Santamaria and Satitsuksanoa, 2022), alongside the balance between repair-stimulating signals such as IL-22 and IL-17 and inflammation-amplifying axes such as IL-23-driven Th17 responses, ultimately determines the functional recovery or continued apoptosis and degradation of the barrier (Konstantinus et al, 2020; Tait Wojno and Artis, 2012). This topology enables the identification of consistent signaling across all levels of biological complexity.

## In vitro models from cellular simplicity to physiological complexity

### Simplified epithelial monolayers

Simplified epithelial monolayers are the most widely used models for dissecting specific aspects of intestinal barrier function under controlled conditions. Caco-2, T84, and HT-29 cell lines differentiate into polarized monolayers on permeable Transwell inserts, allowing independent manipulation of apical and basolateral compartments. The compartmentalized nature of this system makes it possible to investigate how microbiota-derived metabolites applied apically can transition across the barrier to modulate immune cells in the basolateral chamber. This setup allows for the study of complex epithelial-immune crosstalk, where immune cells are impacted by the microbial environment on the epithelium to regulate barrier integrity or inflammatory signaling (Adams et al, 2024).

However, conclusions drawn from these systems must be strictly stratified, as their malignant origin imposes significant metabolic and signaling biases that deviate from normal intestinal physiology (Galeano Nino et al, 2022). Unlike primary colonocytes that rely on mitochondrial oxidative phosphorylation, these cancer-derived lines exhibit a Warburg-like glycolytic metabolism (Bao et al, 2026). For instance, in normal cells, butyrate is rapidly oxidized as a primary energy source. However, the glycolytic state in Caco-2 cells prevents efficient oxidation, causing butyrate to accumulate in the nucleus as a histone deacetylase (HDAC) inhibitor and trigger stress-related transcriptional programs that may not occur in vivo (Fawad et al, 2022). Therefore, claims regarding the transcriptional regulation of TJ protein by butyrate require validation in primary-derived models to ensure they reflect physiological homeostasis rather than a malignant metabolic artifact. Moreover, the lack of functional goblet cells in Caco-2 cells results in a negligible mucus layer and absent epithelial turnover (Floor et al, 2025). Consequently, assessments are restricted to static TEER and TJ protein assays, which overlook the dynamic barrier function and regeneration of the native intestine.

Beyond metabolic biases, these transformed lines often exhibit an aberrant signaling topology. Specifically, cancer-derived cells can overactivate TGR5/cAMP signaling and uncouple FXR-mediated programs from the cell’s differentiation state, leading to abnormal responses to SBAs (Deutschmann et al, 2018). Similarly, while indole metabolites such as IAld and IPA activate AhR- and PXR-driven programs to enhance barrier function in primary cell lines, this signaling is often redirected toward tumor-supportive outputs in immortalized cells (Jia et al, 2024). Consequently, the readouts from these models often reflect the behavior of a malignant phenotype rather than a physiological intestine. Mechanistic conclusions regarding microbial metabolites and inter-compartmental crosstalk should therefore be validated in primary-derived models such as organoid-derived monolayers to ensure they are physiologically representative and to avoid translational misinterpretation.

### Intestinal organoid models

Three-dimensional (3D) intestinal organoids derived from primary human intestinal stem cells have emerged as a superior model to overcome the inherent limitations of immortalized cell lines (Bakker et al, 2022). The organoids maintain the key features of the native epithelium, including mucus-secreting goblet cells, rare enteroendocrine cells, and antimicrobial peptide-secreting Paneth cells (Abud et al, 2024). Importantly, organoids preserve the oxidative phosphorylation-driven metabolism of a healthy primary epithelium, thus avoiding the Warburg-like glycolytic metabolic disorder (Burns and Manda, 2017). This metabolic authenticity ensures that microbial metabolites, such as butyrate, are processed as physiological energy sources rather than purely as epigenetic stress signals (Fei et al, 2025).

However, the closed spherical architecture of traditional organoids presents a significant spatial challenge, as the apical surface is isolated within the organoid core. To study the direct effects of microbial stimuli on the “inside-out” brush border, researchers must employ specialized techniques to bridge the gap between the culture medium and the lumen. Microinjection techniques deliver bacteria or microbial metabolites directly into the sealed lumen of intact organoids via micro-needles (Puschhof et al, 2021). By maintaining the tissue’s 3D structural integrity and oxygen gradients, microinjection is essential for capturing the molecular events of Phase I in a physiological environment. However, its low throughput has led to the development of apical-out organoids as an alternative approach (Shull et al, 2019). By reversing their polarity to expose the luminal surface to the surrounding culture medium, this approach provides a concrete platform for investigating the invasive mechanisms of enteric pathogens like *Salmonella enterica* or the rapid nutrient uptake of microbial metabolites, which are characteristic of barrier failure in Phase II (Co et al, 2019).

Moreover, researchers have increasingly transitioned toward organoid-derived monolayers on Transwell inserts or microfluidic chips, enabling interactions with the underlying mucosal environment (Hofer et al, 2025). These platforms provide critical access to the basolateral surface, facilitating the co-culture of the intestinal epithelium with autologous immune cells or stromal components. For instance, the integration of tissue-resident T cells with autologous organoids has recently enabled the study of how the mucosal immune compartment shapes epithelial responses to the microbiota, effectively circumventing the allogeneic reactions typically observed in mismatched donor models (Recaldin et al, 2024).

By re-establishing these compartmentalized interactions, these techniques treat the organoid as a miniature, accessible human intestine, providing a reliable platform to test how different microbial strains strengthen or damage the barrier under physiologically relevant conditions (Nakamoto et al, 2019). However, limitations such as short culture duration and donor-dependent variability affect their suitability for modeling chronic inflammation, immune responses, and long-term host-microbiota interaction. Specifically, the restricted lifespan of organoid-derived monolayers precludes the study of chronic barrier degradation and the gradual shift in paracellular transport seen in persistent inflammatory states. In addition, significant fluctuations in baseline TJ protein expression and mucus thickness between donors can obscure the reinforcement effects of microbial metabolites, making it difficult to establish reproducible dose-response curves for barrier-protective interventions. Consequently, a multi-layered evaluation that integrates mucus thickness with classical permeability assays is required. More importantly, these organoid platforms are best integrated within a broader in vitro and ex vivo model to fully decode metabolite–host interaction.

### From dynamic fermentation to anaerobic co-culture models

To accurately simulate the intestinal barrier, the model must accommodate the oxygen-sensitive nature of the gut microbiota. Dynamic in vitro fermentation models are designed to analyze complex microbial fermentation, cross-feeding, and metabolic profiles over extended periods under strictly anaerobic conditions (Singh et al, 2022). These multi-compartment systems, such as the Simulator of the Human Intestinal Microbial Ecosystem (SHIME), TNO in vitro model of the stomach and small intestine (TIM-1), and TNO in vitro model of the colon (TIM-2), simulate sequential gastrointestinal compartments with controlled pH, retention times, and nutrient delivery, and inoculate human fecal microbiota, providing a robust framework for defining metabolite profiles that are difficult to reproduce in static cultures (Maas et al, 2023; Zhu et al, 2024). Importantly, the incorporation of mucus-mimicking compartments enables the study of mucosa-associated microbial communities, which are often underrepresented in luminal-only cultures (Bron et al, 2025).

**Figure 2:**
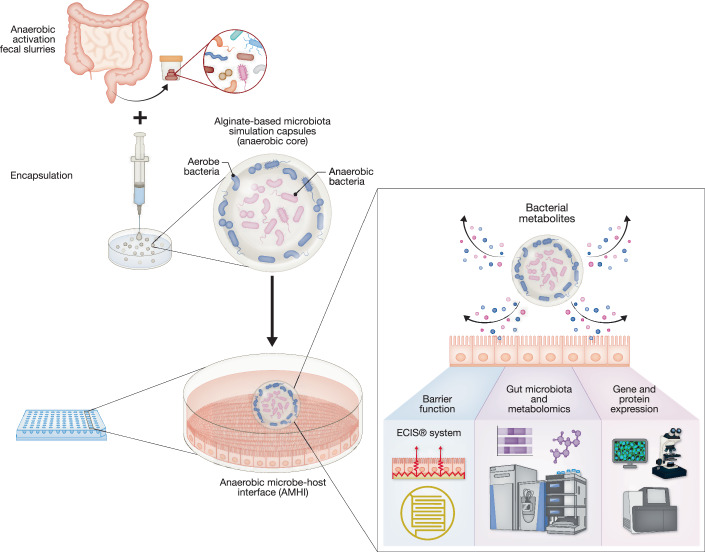
AMHI system: an anaerobic-microbial-host interaction platform. The AMHI system enables the encapsulation of human gut microbiota derived from fecal samples within alginate-based hydrogel beads, which are co-cultured with intestinal epithelial cells under controlled hypoxic conditions. This configuration maintains microbial viability and fermentation activity while restricting direct bacterial-epithelial contact, thereby mimicking key spatial features of the intestinal environment. Functional analyses include epithelial barrier integrity assays, microbial composition profiling, metabolomic assessments, and host gene and protein expression studies, providing an integrated framework for investigating anaerobic host-microbe interactions.

However, because these fermentation models lack an epithelial interface, their readouts must be coupled with co-culture platforms to establish functional relevance. Anaerobic co-culture systems address this by creating a steep oxygen gradient for the survival of both obligate anaerobes and aerobic host cells, enabling microbial fermentation while preserving epithelial viability. One pioneering model is the Human-microbial crosstalk (HuMiX) system, which uses microfluidic separation and integrated oxygen sensors to maintain a strictly anaerobic compartment, demonstrating enhanced TJ integrity and AMPs production under near-physiological conditions (Lucchetti et al, 2024; Shah et al, 2016). Moreover, it captures the dynamic interactions of secreted metabolites between host and microbes (Yang et al, 2024). Alternatively, the encapsulation-based Anaerobic Microbe-Host Interface (AMHI) employs semipermeable hydrogel beads to spatially separate bacteria from the epithelium while allowing for the controlled diffusion of metabolites (Hosseini and Varidi, 2021; Mamidi and Delgadillo, 2021). This system is particularly suited for linking microbial metabolism to epithelial barrier modulation, effectively capturing the transition into Phase II where biochemical signals dominate the host response (Fig. 2).

These systems provide valuable insight into host-microbe interactions, although further optimization is needed to more closely mimic the high-speed flow dynamics and mechanical strain of the native intestine. Incorporating these physical cues may enhance glycocalyx development and support more physiologically relevant tight junction maturation, thereby improving the accuracy of microbial translocation measurements. In addition, the relatively small volumetric capacity of current co-culture chambers highlights an opportunity to refine system design, for example, by improving medium exchange or flow conditions, to better control the accumulation of metabolic byproducts and maintain epithelial homeostasis. This would help to more clearly resolve strain-specific microbial effects.

### Microfluidic “gut-on-a-chip” systems

To address the lack of physical cues in static anaerobic systems, Microfluidic gut-on-a-chip has emerged as an alternative strategy that represents the pinnacle of synthetic integration (Ballerini et al, 2025). By introducing controlled shear stress and peristalsis-like deformation, these platforms promote epithelial polarization, villus-like architecture, TJ maturation, and mucus secretion, thereby enhancing the physiological relevance of epithelial barrier models (Jeon et al, 2022).

A defining advantage of the gut-on-a-chip platform is its ability to sustain a stable oxygen gradient through precise perfusion, allowing live microbial communities to coexist with host cells over extended periods (Liu et al, 2023). In addition, this capability allows dynamic assessment of how microbial metabolites, community composition, and mechanical forces jointly influence barrier integrity and epithelial signaling (Jalili-Firoozinezhad et al, 2019). Uniquely, the gut-on-a-chip can model Phase III by incorporating vascular channels seeded with immune components such as macrophages, dendritic cells, or peripheral blood mononuclear cells, which allows researchers to observe the real-time recruitment of immune cells across the endothelium in response to microbial triggers (Donkers et al, 2024; Maurer et al, 2019).

Although this platform is positioned as an integrative interface, several technical limitations still exist. The absence of neuronal and vascular components makes these models lack the systemic feedback, such as enteric nervous system-mediated mucus secretion or capillary-driven clearance, which may overestimate the direct toxic effects of metabolites on paracellular permeability by neglecting these innate compensatory mechanisms. In addition, inconsistent mechanical stress disturbs the location and expression of TJ proteins, potentially triggering barrier failure to faithfully mimic human peristaltic frequency. Such disruption is a byproduct of physical shear rather than biological signaling. Finally, the current reliance on static TEER and TJ protein assays overlooks the chip’s unique capacity to monitor shear-induced mucus dynamics and epithelial turnover, which may fail to accurately predict epithelial resilience and long-term barrier homeostasis under variable nutrient and flow conditions.

## Ex vivo platforms: preserving the native architecture

Despite the increasing advancement of engineered in vitro systems, ex vivo platforms remain the definitive reference for human intestinal physiology. By utilizing tissues directly isolated from humans or animals, these models preserve the native architecture of the mucosa, including the epithelium, lamina propria, resident immune cells, and elements of the enteric nervous system within their native extracellular matrix.

### Models for capturing the complexity of the intact mucosa

Intestinal explant and mucosal biopsy culture offer an unmatched snapshot of the in vivo state. Intestinal explants preserve whole segments of freshly isolated intestinal tissue, including epithelium, lamina propria, immune cells, and elements of the muscularis, making them ideal for investigating microbial colonization and host defense mechanisms (Sodhi et al, 2021).

Explant models are particularly adept at investigating microbial colonization, host defense mechanisms, and tissue responses under conditions that are physiologically relevant to the in vivo mucosa. For example, colonic mucosa explants exposed to enterotoxigenic *E. coli* exhibit pro-inflammatory cytokine release and can be attenuated by probiotic treatment (Fabrega et al, 2016). This highlights the model’s capacity to recapitulate complex microbe-induced immune responses and evaluate pharmacological or dietary interventions without the need for exogenous immune cell integration. Similarly, mucosal biopsies obtained through minimally invasive endoscopy preserve epithelial polarity and resident immune cells within the lamina propria, allowing for the simultaneous assessment of barrier function and cytokine secretion in patient-specific contexts (Furuta et al, 2024). Biopsies from patients with IBD have been used to directly observe pathogen adherence patterns and the protective effects of probiotics or dietary metabolites on inflammatory activation, which provides a window into disease-specific pathology that synthetic models cannot yet fully replicate (Armstrong et al, 2023). However, the utility of these models is fundamentally constrained by their limited viability (24–48 h) and the technical difficulty of maintaining complex anaerobic microbiota without triggering rapid tissue degradation due to microbial overgrowth (Eslami Amirabadi et al, 2022).

To improve experimental reproducibility and throughput, precision-cut intestinal slices (PCIS) have emerged as a standardized alternative. Fresh human or animal intestine-derived slices preserve epithelial, resident immune cells, stromal, muscles components in situ, enabling the maintenance of coordinated epithelial-immune-stromal interactions (Punyadarsaniya et al, 2015). Currently, PCIS has been applied to studies of epithelial barrier disruption, mucosal immune activation, and functional responses to microbial stimuli, including analyses of pathogen-induced epithelial injury and drug responses targeting tissue remodeling (Grieger et al, 2025). However, its use for direct co-culture with live gut microbiota or fecal consortia remains largely unexplored. Recent innovations, including the integration of PCIS with microfluidic perfusion or oxygen-controlled incubators, have begun to explore host-microbiota interaction (Delong et al, 2024; Grieger et al, 2025). However, the slicing process inherently disrupts the luminal-to-basolateral directionality, allowing microbes to bypass the epithelial barrier via lateral cut edges. This loss of structural polarity, coupled with the presence of residual native microbiota that is difficult to eliminate, can compromise the biological relevance of controlled experimental designs. To mitigate these, researchers should utilize compartmentalized micro-chambers to re-establish directional exposure and employ standardized antibiotic depletion protocols to minimize confounding signals from the endogenous microbiome.

Despite these advances, these models are challenged by tissue viability and degradation after resection. This limited window precludes the long-term observation of epithelial turnover and mucus dynamics, which are critical determinants of barrier resilience that static TEER or protein-based assays cannot fully capture. The most immediate consequence is the apoptosis that triggers paracellular leakage, which may be mistakenly interpreted as a biological response to microbial metabolites rather than a symptom of tissue degradation. In addition, the technical difficulty of maintaining anaerobic conditions often leads to microbial overgrowth, which can artificially induce epithelial stress and UPR activation, thereby confounding the causal inference between specific metabolites and barrier resilience.

### Ussing chamber system for assessing barrier function

The Ussing chamber is a classical ex vivo platform for assessing intestinal barrier function. In this system, freshly isolated segments of intestine or endoscopic biopsy specimens can be mounted between two opposing chambers that independently control the luminal and serosal microenvironments, which enables the real-time measurements of TEER, ion transport, paracellular permeability, and cytokine secretion (Metzler-Zebeli et al, 2022; Vanuytsel et al, 2021). This configuration is indispensable for detecting regional differences in barrier permeability and the distinct impact of inflammatory conditions (Thomson et al, 2019). Furthermore, dietary interventions, including β-glucans supplementation, have been shown to enhance epithelial barrier integrity while simultaneously promoting bacterial adherence, illustrating the value of studying diet-microbe-host interactions (Ewaschuk et al, 2012). Recent advances have introduced the mini-Ussing chamber system that reduces tissue requirements and extends viability, reinforcing the platform’s role in linking microbial metabolic processing to functional host readouts (Kondo and Miyake, 2023).

However, the Ussing chamber is fundamentally limited by the absence of systemic circulation, which leads to rapid oxygen consumption and metabolite accumulation. Such issues can artificially trigger oxidative stress, potentially masking the protective or damaging effects of microbial metabolites (Poeta et al, 2023). Furthermore, neural control of mucus secretion and ion transport was disrupted during the tissue removal, leading to overestimation of ex vivo readouts of barrier vulnerability (Edwinson and Grover, 2019). Future studies should prioritize the use of mini-Ussing chambers and oxygenated physiological buffers to preserve goblet cell activity, ensuring that TEER readouts are contextualized within the tissue’s defense capacity.

### Impact of tissue orientation and imaging depth

A critical factor that is frequently overlooked in ex vivo research is the spatial orientation of the tissue during sample collection. Unlike 2D monolayers, ex vivo explants and PCIS possess significant structural depth, which fundamentally dictates what biological events are captured as the imaging direction of the microscope objective focuses from the mucosal or serosal side.

This orientation is particularly decisive when modeling the early stages of Phase I. For example, imaging exclusively from the serosal side creates a significant blind area when tracking the translocation of bacteria labeled with fluorescent (Fan et al, 2025). Due to the thick layers of the muscularis and submucosa acting as optical barriers, the microscope may fail to detect the initial thinning of the apical mucus layer or the early “leaks” in TJ that characterize Phase II. When the signal is detected from the serosal side, the barrier has already undergone the collapse, leading to an underestimation of the protective effects of early interventions such as probiotics or SCFAs

Furthermore, the physical mounting of these tissues in specialized devices introduces potential mechanical artifacts. In Ussing chambers, the mechanical pressure required to secure the tissue can cause edge damage, creating paracellular pathways for ions and markers that mimic Phase I (Krug et al, 2025). Therefore, researchers must standardize the luminal-to-basolateral axis, explicitly report the focal depth of imaging, and the imaging direction. Only by ensuring that the observation starts from the mucosal surface can we accurately distinguish between a true biological response to microbial metabolites and technical artifacts caused by the loss of spatial integrity.

## Clinical translation of in vitro and ex vivo models

The ultimate value of in vitro and ex vivo models lies in their ability to recapitulate human disease relevance and predict clinical translation. By integrating microbial signaling with host-specific genetic or inflammatory backgrounds, these models have become indispensable for studying the impact of barrier function.

### IBD

IBD serves as the primary clinical disease for intestinal barrier failure (Neurath et al, 2025). While animal models often fail to capture the nature of IBD in humans, patient-derived organoids and biopsy cultures preserve the individual’s susceptibility to inflammation. Notably, the biological context of IBD fundamentally shifts metabolite signaling. For instance, SCFAs and indoles reinforce barrier integrity under homeostatic conditions, but this protective capacity is often attenuated in the pro-inflammatory environment of IBD due to the downregulation of receptors such as AhR or GPR43 (Bernardi et al, 2024). These models have illustrated that IBD-associated dysbiosis directly damages junctional integrity in Phase I and promotes immune activation in Phase III. Furthermore, gut-on-a-chip platforms that incorporate peristaltic strain and perfusion have revealed that mechanical forces can either exacerbate or mitigate cytokine-induced barrier damage, providing a mechanistic basis for intestinal inflammation in patients under static or non-static conditions.

### Colorectal cancer

In colorectal cancer, the interaction between microbes and the epithelial barrier shifts from maintaining homeostasis to tumorigenesis (Yu et al, 2012). Advanced co-culture models, such as colorectal cancer-organoids, are essential to demonstrate how specific microbes drive this progression. For example, *pks+ Escherichia coli* promotes tumorigenesis by releasing colibactin, which induces double-strand DNA breaks in host cells (Pleguezuelos-Manzano et al, 2020). Importantly, these advanced models demonstrate a metabolic reversal in signal transduction. In physiological conditions, indoles reinforce TJ and suppress inflammation responses. However, due to the Warburg effect, colorectal cancer cells alter their response to indoles, shifting from promoting barrier integrity to modulating tumor-driven immune responses (Trejo et al, 2026). This underscores how the malignant metabolic state redefines the functional outcome of microbial metabolites, a distinction that is lost in generalized homeostasis models.

### Neurodegenerative diseases

One of the most widely used applications of neurodegenerative diseases is the study of the gut-brain axis in Parkinson’s disease and Alzheimer’s disease. Microbiome-derived signals, particularly SCFAs and amyloid-like proteins, can influence systemic inflammation and blood-brain barrier (BBB) integrity (Fock and Parnova, 2023). At present, by coupling gut-on-a-chip devices with BBB-on-a-chip through microfluidic circuits, researchers can monitor the sequential transit of microbial metabolites from the intestinal lumen to the circulatory system and finally to the neural system (Pizarroso et al, 2021). These multi-organ-chip systems have identified specific microbial consortia that trigger α-synuclein misfolding in enteroendocrine cells, providing a potential signal for neurodegeneration long before clinical symptoms appear.

### Drug discovery and precision medicine

These platforms are providing high-fidelity strength to revolutionize drug development for individual patients. PCIS and mini-Ussing chambers are being used to screen the barrier-protective effects of novel postbiotics and engineered microbial therapeutics (Mosca et al, 2022). Moreover, these models maintain the patient’s unique receptor, such as AhR distribution, to predict which individuals will respond to specific dietary interventions or biological therapies, thereby reducing the high failure rate of traditional clinical trials (Dinallo et al, 2019).

To sum up, the clinical translation of these models hinges on their ability to account for the context-dependent nature of microbial signaling. In IBD and colorectal cancer, the intestinal barrier’s response to metabolites such as SBAs and SCFAs is not uniform but is explicitly dictated by the host’s inflammatory state and cellular metabolic profile. Therefore, explicitly qualifying mechanistic claims within these specific biological contexts is essential for moving beyond idealized homeostasis toward a precise understanding of human disease.

## Conclusion and future perspectives

The study of the intestinal barrier has evolved from observing a static physical barrier to decoding a dynamic molecular interaction between microbes and host cells. As evaluated throughout this review, microbial metabolites such as SCFAs, SBAs, and tryptophan derivatives do not simply maintain the barrier; they regulate a sequential lifecycle of protection and decay through specific receptors and pathways. However, the recurring challenge in this field is the translational gap caused by experimental designs that fail to match the biological phase being studied (Table 2). Therefore, the focus of future research must shift from seeking a perfect model to adopting a context-dependent integrative strategy, as no single platform can replicate the full biological complexity of the human intestine.

**Table 2:** Comparison of in vitro and ex vivo models.

Type	Model	Advantages	Limitations	Applications	Key readouts
In vitro	Simplified epithelial monolayers (Caco-2, T84, HT-29-MTX)	High control, reproducible; quantitative TEER and permeability readouts; scalable	Lack of immune and stromal cells; oversimplified mucus and oxygen gradients	High-throughput screening of defined metabolites, postbiotics, and receptor agonists	TEER, FITC-dextran permeability, ZO-1, claudin, occludin, mucin staining, cytokines release
In vitro	Anaerobic co-culture systems (HuMiX)	Separate oxygen control; obligate anaerobe co-culture; real-time monitoring	Short co-culture duration; limited microbial diversity; no immune cells	Studying anaerobe-induced barrier reinforcement and metabolite production	Real-time TEER, oxygen monitoring, AMPs, transcriptomics, metabolite profiling
In vitro	Anaerobic co-culture systems (AMHI)	Create hypoxic microenvironment; slow metabolite release; prevent bacterial overgrowth	Variable diffusion rates; no direct adhesion and invasion study	Linking microbial fermentation to barrier protection; personalized microbiota responses	ECIS (dynamic TEER), SCFA, mucin-related gene expression
In vitro	Organoid-microbiota interaction system (Microinjection systems)	Preserve 3D polarity; precise luminal delivery; immune co-culture possible	Low throughput; short-term viability	Studying early infection events, probiotic colonization, immune-epithelial crosstalk	Real-time imaging, pathogen growth kinetics, TJ dynamics, IL-1β-dependent cell death
In vitro	Dynamic in vitro fermentation models (SHIME, TIM-1, TIM-2)	Maintain complex microbiota; region-specific fermentation; mucus layer	No host cells; need coupling with epithelial and immune models	Prebiotic screening, studying microbial metabolites, donor-specific microbiota	SCFA, bile acids, indole derivatives, gas production, pH, metagenomics
In vitro	Microfluidic ‘Gut-on-a-chip’ systems	Flow, shear, oxygen gradients; long-term host-microbe coexistence; real-time imaging	Technically demanding; immune and neuronal elements still limited	Modeling dynamic host-microbe interactions, probiotic intervention	TEER, permeability, live imaging, cytokines, villus height, mucus thickness
Ex vivo	Intestinal explants & Mucosal biopsy culture	Include immune and stromal cells; patient-specific phenotypes	Short viability (24–48 h); low throughput	Validating in vitro findings, integrated immune-epithelial responses	TEER, cytokine secretion, transcriptomics, pathogen adherence imaging
Ex vivo	Ussing chamber systems (classical, mini)	Gold standard for transport physiology; quantitative electrophysiology	Fresh tissue required; short-term only	Barrier permeability measurement, ion transport studies	TEER, ion flux, paracellular permeability, cytokine release
Ex vivo	Precision-cut intestinal slices (PCIS)	Preserve multicellular architecture; reproducible thickness	Limited viability (24-96 h); hard to co-culture with complex microbiota	Pathogen invasion, fibrosis, drug testing	Cytokines (IL-6, TNF-α), fibrosis markers (collagen, α-SMA), histology and immunohistochemistry

Current evidence suggests that while immortalized cell lines offer high-throughput screening, their malignant “Warburg” metabolism and incomplete receptor location often lead to a fundamental misinterpretation of metabolite signaling. To this end, researchers should increasingly prioritize 3D organoids, anaerobic co-culture systems, and gut-on-a-chip systems to capture the metabolic and mechanical profiles required for studying junctional remodeling in Phase I and mucus dynamics in Phase II. Furthermore, these findings must be validated in ex vivo platforms to capture the immune-driven permeability in Phase III at the tissue level in diseases like IBD and colorectal cancer.

However, a critical evaluation of current technologies suggests that while in vitro and ex vivo systems have evolved significantly, they have not yet fully recapitulated the systemic complexity of the in vivo environment. Most existing models remain reductionist, typically focusing on a limited subset of immune cells or simplified microbial consortia. A major technical hurdle in these immunocompetent systems is the allogeneic reaction caused by major histocompatibility complex (MHC) incompatibility when combining epithelial and immune cells from different donors. These non-physiological immune responses can mask the true regulatory effects of microbial metabolites, representing a significant bottleneck for traditional co-culture platforms (Recaldin et al, 2024). Moreover, they often lack the integrated systemic circulation, the full repertoire of the mucosal immune system, and the enteric nervous system that collectively govern barrier homeostasis in vivo. Therefore, these systems should be viewed as high-fidelity mechanistic platforms rather than complete replicas of the human intestine.

**Figure 3:**
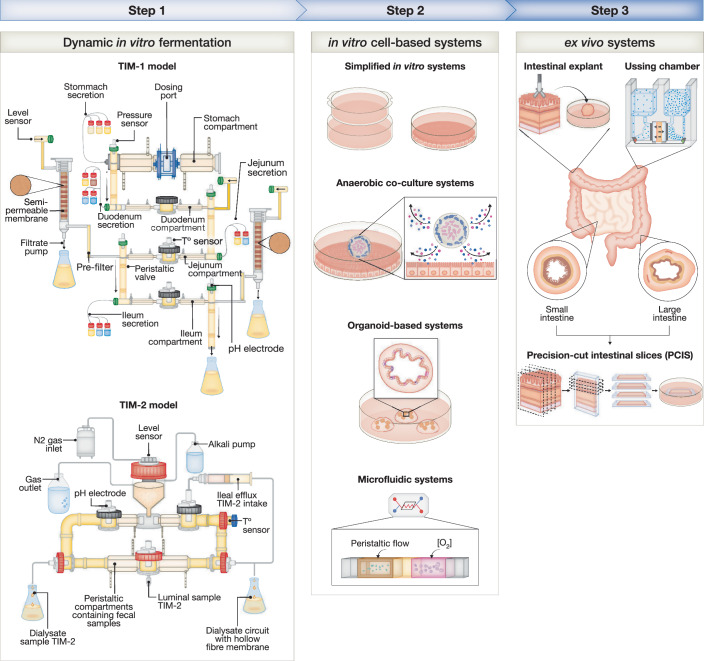
The stepwise and integrative strategy for model selection. The workflow with three sequential steps illustrates a transition from model optimization to context-dependent application. Step 1: Dynamic in vitro fermentation models such as TIM-1 and TIM-2 are utilized to define microbial metabolite profiles under controlled dietary or microbial conditions. Step 2: Identified metabolites are mechanistically interrogated using in vitro cell-based systems ranging from simplified monolayers and anaerobic co-cultures to organoid-based and microfluidic gut-on-a-chip systems to resolve receptor activation, barrier function, and epithelial signaling pathways. Step 3: Key findings are validated in ex vivo systems, including intestinal explants, Ussing chambers, and precision-cut intestinal slices (PCIS) to capture multicellular immune interactions and disease-relevant phenotypes, thereby maximizing human relevance.

Future research should switch the principle of model selection from model optimization to context dependence and develop stepwise and integrative strategies (Fig. 3). Firstly, dynamic in vitro fermentation models can be used to define microbial metabolic profiles under controlled dietary or microbial conditions. Then, identified metabolite profiles can be mechanistically interrogated in anaerobic co-culture systems, organoid-derived, and microfluidic gut-on-a-chip systems to resolve receptor activation, barrier function, and epithelial signaling pathways. Finally, key findings should be validated in ex vivo tissues to capture multicellular immune interactions and disease-relevant phenotypes. Such a multidirectional strategy provides a rational framework for minimizing overinterpretation while maximizing human relevance.

Looking forward, progress in the field will focus on establishing standardized multi-model workflows that integrate metabolic profiling, epithelial biology, and tissue-level immune responses instead of developing more complex individual models. Combining such strategies with patient-derived tissues, multi-omics, and computational modeling will be critical for translating microbiota research into personalized treatments. Ultimately, aligning experimental design with biological complexity represents a necessary step toward clinically meaningful insights into microbiota-host interactions.

## Pending issues

Developing donor-matched systems to eliminate MHC-driven immune interference.

Establishing universal stepwise workflows that combine a dynamic in vitro fermentation, in vitro cell-based systems, and ex vivo validation.

Integrating neural components into in vitro models to explore how the brain regulates mucus secretion and barrier homeostasis in response to microbial cues.

Further investigating how the malignant or inflammatory environment, such as IBD or CRC, fundamentally flips the functional role of metabolites from protective to toxic.

Using coupled-chip systems to track the dynamic movement of metabolites across the gut and blood–brain barrier.

## Supplementary information

Peer Review File

## Glossary

Blood-brain barrier (BBB): A highly selective semipermeable border that protects the brain by strictly controlling which substances or cells can enter from the bloodstream.
MHC Incompatibility: A biological mismatch that occurs when cells from two different individuals are mixed, causing an “allogeneic reaction” that can ruin experimental results in complex gut models.
Microbial Metabolites: Small molecules produced by gut bacteria during the fermentation of food, which act as messengers to signal the host’s cells to maintain or repair the intestinal barrier.
Microbial-associated molecular patterns (MAMPs): Highly conserved and essential structural molecules on microorganisms that allow host cells to identify them as either beneficial or pathogenic, determining the innate immune response.
Proteostasis (UPR): The cellular system that ensures proteins are synthesized, folded, and degraded correctly. When this system is overwhelmed by stress, it serves as an early warning sign of intestinal barrier failure.
Warburg Effect: A metabolic shift commonly seen in cancer cells where they favor sugar fermentation over oxygen-based energy production; in research, this can cause lab-grown cells to react incorrectly to bacterial signals.
