# Supplementary material for: Experimental models for intestinal host-microbe interactions
Source: EMBO Mol Med. 2026 May 21;18(7):2533–46. doi: 10.1038/s44321-026-00450-4 (PMC13365559; doi:10.1038/s44321-026-00450-4)
Supplement: Supplementary file 1 — Peer Review File [file 44321_2026_450_MOESM1_ESM.pdf]

EMBO Molecular Medicine

Experimental Models for Intestinal Host-Microbe Interactions

Yuqi Li, Naschla Gasaly, and Paul Vos

Corresponding author(s): Yuqi Li (yuqi.li@maastrichtuniversity.nl) , Paul Vos (paul.devos@maastrichtuniversity.nl)

Review Timeline:

Submission Date:	6th Mar 26
Editorial Decision:	27th Mar 26
Revision Received:	10th Apr 26
Editorial Decision:	23rd Apr 26
Revision Received:	23rd Apr 26
Accepted:	27th Apr 26

Editor: Zeljko Durdevic

Transaction Report:

(Note: With the exception of the correction of typographical or spelling errors that could be a source of ambiguity, letters and reports are not edited. Depending on transfer agreements, referee reports obtained elsewhere may or may not be included in this compilation. Referee reports are anonymous unless the Referee chooses to sign their reports.)

27th Mar 2026

Dear Dr. Li,

Thank you for the submission of your manuscript to EMBO Molecular Medicine. We have now received feedback from the two reviewers who agreed to evaluate your manuscript. As you will see from the reports below, the referees are overall positive about timeliness and interest of the topic; however, they also raise serious concerns that should be addressed in a major revision of the current manuscript.

Further consideration of a revision that addresses reviewer's concerns in full will entail an additional round of review. Acceptance or rejection of the manuscript will depend on the completeness of your responses included in the next, final version of the manuscript. For this reason, and to save you from any frustrations in the end, I would strongly advise against returning an incomplete revision.

I hope that the referees' comments do not prove too problematic to address and I look forward to reading your next version.

Yours sincerely,

Zeljko Durdevic

Zeljko Durdevic
Senior Editor
EMBO Molecular Medicine

*** IMPORTANT INFORMATION ***

When submitting your revised manuscript, please include:

1) a .doc formatted version of the manuscript text (including Figure legends and tables)
2) Separate figure files
3) a letter INCLUDING the reviewer's reports and your detailed responses to their comments.

Also, and to save some time should your paper be accepted, please read below for additional information regarding some features of our research articles:

1) Glossary: EMBO Molecular Medicine articles will be accompanied by a glossary explaining some of the terms used for laymen. I identified the following:

_____, _____, _____

Could you please help us in identifying terms that may need an "explanation" other terms that we can add to the glossary.

2) Pending issues: At the end of each article we will have a box highlighting issues that still need further studies and where research efforts should converge (we call this the Pending issues box). From my reading I would say:

----

but I can see there may be many more. Could you work on this as well?

3) Disclosure and competing interest statement: Please include a statement declaring any competing commercial interests in relation to your submitted work.

4) Please note that we now mandate that all corresponding authors list an ORCID digital identifier. This takes <90 seconds to complete. We encourage all authors to supply an ORCID identifier, which will be linked to their name for unambiguous name identification.

Currently, our records indicate that there is no ORCID associated with your account.

Please click the link below to provide an ORCID:
Link Not Available
-

Thank you,

Zeljko Durdevic

Zeljko Durdevic
Editor
EMBO Molecular Medicine

***** Reviewer's comments *****

Referee #1 (Remarks for Author):

Overall, the review provides a useful overview of current in vitro and ex vivo host-microbe interaction models. However, it appears to lack a clear discussion of one of the key assumptions introduced in the manuscript: whether in vitro and ex vivo systems have evolved sufficiently to recapitulate the complexity of in vivo models when considering the microbiome, intestinal epithelium and immune system simultaneously.

This point should be addressed more explicitly in my opinion. A critical evaluation of how close current models are to reproducing the complexity of in vivo systems would significantly strengthen the manuscript and provide readers with a clearer perspective on future research directions, current limitations and realistic expectations for these technologies.

Furthermore, the literature includes examples of advanced in vitro and ex vivo models that attempt to incorporate immune components into microbiome-epithelium systems. Including and discussing these studies would improve the completeness of the review and better support the central theme of the manuscript.

Suggested references to include

Recaldin, T., Steinacher, L., Gjeta, B. et al. Human organoids with an autologous tissue-resident immune compartment. Nature 633, 165-173 (2024). https://doi.org/10.1038/s41586-024-07791-5

Puschhof, J., Pleguezuelos-Manzano, C., Martinez-Silgado, A. et al. Intestinal organoid cocultures with microbes. Nat Protoc 16, 4633-4649 (2021). https://doi.org/10.1038/s41596-021-00589-z

Galeano Niño, J.L., Wu, H., LaCourse, K.D. et al. Effect of the intratumoral microbiota on spatial and cellular heterogeneity in cancer. Nature 611, 810-817 (2022). https://doi.org/10.1038/s41586-022-05435-0

Ballerini, M., Galiè, S., Tyagi, P. et al. A gut-on-a-chip incorporating human faecal samples and peristalsis predicts responses to immune checkpoint inhibitors for melanoma. Nat. Biomed. Eng 9, 967-984 (2025). https://doi.org/10.1038/s41551-024-01318-z

Michelle Maurer, et al A three-dimensional immunocompetent intestine-on-chip model as in vitro platform for functional and microbial interaction studies. Biomaterials, Volume 220, 2019, 119396, https://doi.org/10.1016/j.biomaterials.2019.119396.

Hou, Q., Ye, L., Liu, H. et al. Lactobacillus accelerates ISCs regeneration to protect the integrity of intestinal mucosa through activation of STAT3 signaling pathway induced by LPLs secretion of IL-22. Cell Death Differ 25, 1657-1670 (2018). https://doi.org/10.1038/s41418-018-0070-2

Nakamoto, N., Sasaki, N., Aoki, R. et al. Gut pathobionts underlie intestinal barrier dysfunction and liver T helper 17 cell immune response in primary sclerosing cholangitis. Nat Microbiol 4, 492-503 (2019). https://doi.org/10.1038/s41564-018-0333-1

These studies could support the discussion on integrating immune components and microbiota in advanced in vitro systems and help frame future perspectives.

Specific remarks
Introduction
It would be useful to briefly clarify why in vivo models are not considered within the scope of this review. A short justification in the Introduction would help readers better understand the focus on in vitro and ex vivo systems and the rationale behind this choice.

Section 2.3: In this section, it would be helpful to include a practical example to better illustrate the challenges discussed. For instance, the difficulty of studying in vitro host-microbe interactions while simultaneously incorporating adaptive immune systems and 2D or 3D epithelial cultures could be highlighted. In particular, allogeneic reactions caused by MHC incompatibility represent a major limitation when combining immune cells and epithelial models from different donors, and this example would strengthen the discussion.

Section 3.1: It should also be noted that transwell models allow the study of how microbiota-derived metabolites and microorganisms themselves influence epithelial cells and the immune system in the apical and basolateral compartment medium. These systems make it possible to investigate how immune cells conditioned by the microbiome in the basal chamber of the transwell affect epithelial-immune crosstalk. Clarifying these aspects would improve the completeness of the review.

Section 3.3: It would be beneficial to provide more specific examples regarding the possibility of generating apical-out organoids or basal organoid co-cultures. Concrete examples would help readers better understand the practical applications and experimental potential of these approaches.

It would be valuable to include a dedicated section on practical applications of in vitro co-culture systems in biomedical research such as cancer, inflammatory diseases and neurodegenerative diseases

This would help connect the technological developments described in the review with real-world biomedical applications and increase the overall impact of the manuscript.

Referee #2 (Remarks for Author):

The topic is timely and the central question is of genuine importance to the field. However, two recent comprehensive reviews (McCoy et al., 2024; Cheng et al., 2025) have already surveyed the landscape of experimental models for intestinal host-microbe interactions in considerable depth. In its current form, this manuscript does not sufficiently differentiate itself from that existing literature. The proposed stepwise framework linking fermentation, in vitro, and ex vivo systems is conceptually reasonable, but it remains largely descriptive. It does not define how biological signals are transformed across model systems, nor does it identify conserved variables that would enable predictive translation between them. Consequently, the manuscript does not resolve the central problem it articulates: the gap between mechanistic insight and human relevance. Substantial revision is required before this work can be considered for publication.
Major comments
1. The title implies a synthesis spanning epithelial biology broadly, yet the analysis is confined to intestinal systems. The authors must either restrict the stated scope explicitly to gut barrier biology throughout the manuscript, or incorporate mechanistic coverage of other epithelial tissues with comparable depth. Current claims of generalizable epithelial regulation are not supported by the evidence presented.
2. The stepwise framework assumes continuity across model systems but does not define it. Fermentation systems quantify metabolite outputs (SCFAs, secondary bile acids, indoles); epithelial models report TEER, junctional proteins, and cytokines; ex vivo systems capture integrated tissue responses. These readouts are not mechanistically aligned, and this creates an unresolved break in causal inference. The authors should introduce conserved mechanistic axes that can be tracked across all three platforms. Appropriate candidates include proteostasis stress (e.g., UPR activation), redox state, and cytokine network topology rather than individual cytokines.
3. The manuscript repeatedly frames barrier integrity in terms of reinforcement or disruption. This binary framing obscures the temporal sequence through which barrier failure actually unfolds: an early phase of junctional remodeling and cytoskeletal tension changes; an intermediate phase of mucus layer thinning and increased microbial proximity; and a late phase of immune

activation and cytokine-driven permeability amplification. Individual model systems capture discrete phases of this progression, but the manuscript does not map systems onto these transitions. Doing so would substantially strengthen the integrative value of the framework.

4. While the limitations of in vitro immune modeling are acknowledged, the manuscript does not address how immune activation arises mechanistically from epithelial-metabolite interactions. Receptor-level inputs (GPRs, FXR, AhR) are described in isolation, but their convergence onto shared regulatory nodes is never defined. This represents a significant gap in the mechanistic argument.

5. The limitations of individual platforms should be explicitly linked to their impact on gut integrity studies. For gut-on-a-chip systems, constraints including the absence of neuronal and vascular components, variable nutrient absorption, inconsistent mechanical stress profiles, and interspecies differences are noted but their implications for tight junction regulation, paracellular transport, and epithelial resilience are underexplored. Similarly, organoid systems face donor-dependent variability and short culture duration; the effects of these constraints on reproducibility and long-term modeling of barrier dysfunction should be clarified. Listing limitations without connecting them to specific functional consequences limits the utility of this section for readers designing experiments.

6. Current assessments of gut integrity in this review focus on TEER and tight junction proteins. However, mucus thickness, composition, goblet cell differentiation, and epithelial turnover are equally critical determinants of barrier function and are largely overlooked. The manuscript should address whether in vitro and ex vivo models recapitulate mucus dynamics and how these measures complement TEER and protein-based assays to provide a more complete picture of barrier integrity.

7. Intact tissue platforms such as explants and biopsies preserve native architecture, but systems like precision-cut intestinal slices disrupt the luminal-to-basolateral directionality of microbiota exposure, which compromises biological relevance. Additionally, residual native microbiota is difficult to eliminate in these systems, complicating controlled experimental design. These orientation and microbiome-related pitfalls should be explicitly highlighted when intact tissue models are discussed in the context of barrier function studies.

8. This is a significant concern. Cancer-derived lines (Caco-2, HT-29, HCT116) exhibit glycolytic bias, altered polarity, and aberrant junctional organization that reshape ligand handling and signaling topology in ways that are not representative of normal intestinal epithelium. Specifically, these systems can exaggerate TGR5-cAMP signaling and uncouple FXR programs from differentiation states. Primary systems, including non-transformed IEC lines and organoids, reveal context-dependent responses that diverge substantially. Across metabolite classes, these differences are mechanistically meaningful and not interchangeable: butyrate is rapidly oxidized in differentiated colonocytes, limiting nuclear HDAC inhibitor accumulation, whereas metabolic rewiring in cancer-derived cells enables this effect; secondary bile acids produce dose-dependent barrier-compatible signaling in normal systems but exaggerated toxicity responses in transformed lines; indole metabolites activate coordinated AhR/PXR programs in primary epithelial systems, whereas cancer-derived lines produce incomplete barrier gene induction or tumor-supportive outputs. Conclusions throughout the manuscript must be stratified accordingly.

9. Secondary bile acids are potent TGR5 agonists, whereas FXR is preferentially activated by primary bile acids (e.g., CDCA). The statement that secondary bile acids activate "GPCRs such as TGR5 and FXR" is incorrect and should be revised. Furthermore, secondary bile acid signaling is biphasic: lower concentrations support barrier-compatible signaling, whereas higher concentrations induce membrane and mitochondrial stress, increasing permeability and driving inflammatory signaling. This dose-response transition is entirely absent from the current discussion and represents a mechanistically important omission. Similarly, butyrate is presented as a uniform HDAC inhibitor without accounting for the metabolic state of the cell, which determines whether nuclear accumulation occurs at all. Claims regarding tight junction transcriptional regulation by butyrate require primary evidence from defined, physiologically relevant systems.

10. The manuscript does not distinguish between homeostatic epithelial regulation and disease states such as IBD or colorectal cancer. This distinction is critical, as SCFA, bile acid, and indole signaling diverge substantially across these contexts. Clarification of biological context is required throughout the manuscript, and mechanistic claims should be explicitly qualified by the state in which they apply.

11. Indole-derived metabolites (IPA, IAld) are presented as uniformly barrier-enhancing. In primary epithelial systems, these metabolites activate AhR (IAld preferentially) and PXR (IPA preferentially), inducing coordinated transcriptional programs that strengthen barrier integrity. In cancer-derived systems, this signaling bifurcates into either incomplete barrier gene induction without restored polarity, or into tumor-supportive outputs. This divergence is mechanistically significant and should be explicitly incorporated into the discussion of indole biology.

12. Several mechanistic claims rely on reviews or model systems that do not support the conclusions drawn. Specifically: the butyrate-tight junction claims are not supported by the cited review; Reference 11 (ZeXieYin formula study) cannot isolate secondary bile acid effects due to the presence of multiple confounding variables; Reference 14 relies on tumorigenic Caco-2/HT-29 co-culture yet the conclusions are generalized beyond this context. The citation framework should be rebuilt using primary studies with clearly defined and physiologically appropriate model systems.

13. Structural determinants of microbial signaling, including LPS chemotypes and flagellin variants, shape immune activation thresholds in ways that are directly relevant to barrier regulation. Ligands described as "silent" with respect to NF-kB activation are indicated in Figure 1 but are never mechanistically explained. This limits the manuscript's ability to fully integrate microbial sensing with barrier regulation, which is stated as a core aim.

14. GPCR and nuclear receptor signaling are discussed throughout without defining receptor distribution across intestinal epithelial and immune cell compartments. Because receptor expression determines signaling outcome, this information is essential to the mechanistic arguments being made. The authors should provide a structured mapping of receptor expression across relevant cell types, ideally as a tabulated summary, to anchor the signaling claims made throughout the manuscript.

Minor comments

The authors should ensure figure legends are consistent with the mechanistic content of the main text, particularly Figure 1, where structural ligand categories are depicted but not explained in the accompanying discussion.

Referee #1 (Remarks for Author):

Overall, the review provides a useful overview of current in vitro and ex vivo host-microbe interaction models. However, it appears to lack a clear discussion of one of the key assumptions introduced in the manuscript: whether in vitro and ex vivo systems have evolved sufficiently to recapitulate the complexity of in vivo models when considering the microbiome, intestinal epithelium and immune system simultaneously.

This point should be addressed more explicitly in my opinion. A critical evaluation of how close current models are to reproducing the complexity of in vivo systems would significantly strengthen the manuscript and provide readers with a clearer perspective on future research directions, current limitations and realistic expectations for these technologies.

Response:

We thank the reviewer for this comment. We agree that it is essential to explicitly evaluate how closely current *in vitro* and *ex vivo* models approach the *in vivo* complexity. We have now addressed this hopefully more clearly in Section 6. Firstly, we have added a critical assessment acknowledging that while these models have evolved, they remain high-fidelity reductionist platforms rather than full replicas of the human intestine. In addition, we explicitly discuss the current limitations in simultaneously recapitulating the microbiome, epithelium, and immune system, noting the absence of systemic circulation, the full immune repertoire, and enteric nervous system integration in most platforms. By defining these boundaries, we provide a clearer perspective on future research directions that move from model optimization toward systemic integration through stepwise and integrative strategies. We believe this addition provides the balanced and transparent perspective suggested by the reviewer, ensuring readers have realistic expectations for these technologies.

We have made a modification as follows: "However, a critical evaluation of current technologies suggests that while *in vitro* and *ex vivo* systems have evolved significantly, they have not yet fully recapitulated the systemic complexity of the *in vivo* environment... Therefore, these systems should be viewed as high-fidelity mechanistic platforms rather than complete replicas of the human intestine."

Furthermore, the literature includes examples of advanced in vitro and ex vivo models that attempt to incorporate immune components into microbiome-epithelium systems. Including and discussing these studies would improve the completeness of the review and better support the central theme of the manuscript.

Suggested references to include

Recaldin, T., Steinacher, L., Gjeta, B. et al. Human organoids with an autologous tissue-resident immune compartment. Nature 633, 165-173 (2024). https://doi.org/10.1038/s41586-024-07791-5

Puschhof, J., Pleguezuelos-Manzano, C., Martinez-Silgado, A. et al. Intestinal organoid

cocultures with microbes. Nat Protoc 16, 4633-4649 (2021). https://doi.org/10.1038/s41596-021-00589-z

Galeano Niño, J.L., Wu, H., LaCourse, K.D. et al. Effect of the intratumoral microbiota on spatial and cellular heterogeneity in cancer. Nature 611, 810-817 (2022). https://doi.org/10.1038/s41586-022-05435-0

Ballerini, M., Galiè, S., Tyagi, P. et al. A gut-on-a-chip incorporating human faecal samples and peristalsis predicts responses to immune checkpoint inhibitors for melanoma. Nat. Biomed. Eng 9, 967-984 (2025). https://doi.org/10.1038/s41551-024-01318-z

Michelle Maurer, et al A three-dimensional immunocompetent intestine-on-chip model as in vitro platform for functional and microbial interaction studies. Biomaterials, Volume 220, 2019, 119396, https://doi.org/10.1016/j.biomaterials.2019.119396.

Hou, Q., Ye, L., Liu, H. et al. Lactobacillus accelerates ISCs regeneration to protect the integrity of intestinal mucosa through activation of STAT3 signaling pathway induced by LPLs secretion of IL-22. Cell Death Differ 25, 1657-1670 (2018). https://doi.org/10.1038/s41418-018-0070-2

Nakamoto, N., Sasaki, N., Aoki, R. et al. Gut pathobionts underlie intestinal barrier dysfunction and liver T helper 17 cell immune response in primary sclerosing cholangitis. Nat Microbiol 4, 492-503 (2019). https://doi.org/10.1038/s41564-018-0333-1

These studies could support the discussion on integrating immune components and microbiota in advanced in vitro systems and help frame future perspectives.

Response:

We have incorporated all the suggested references into the revised manuscript to provide a more comprehensive and forward-looking overview.

Specific remarks

Introduction

It would be useful to briefly clarify why in vivo models are not considered within the scope of this review. A short justification in the Introduction would help readers better understand the focus on in vitro and ex vivo systems and the rationale behind this choice.

Response:

We have done this now more clearly. We have modified Section 1. Introduction to help readers to understand better the rationale for focusing on *in vitro* and *ex vivo* platforms. Here we have added a dedicated paragraph to justify our exclusion of *in vivo* models. Specifically, we highlight that while animal models are still valuable, they often exhibit significant interspecies divergences in microbiota composition, mucosal physiology, and immune signaling that limit their direct translational value to humans. Furthermore, we clarify that the

primary objective of this review is to evaluate human-based systems that offer higher tissue accessibility and experimental control for mechanistic studies.

We have revised the text as follows: "The rationale for focusing on *in vitro* and *ex vivo* systems stem from the inherent limitations of current in vivo approaches. ··· In this review, we prioritize human-based in vitro and ex vivo platforms to critically evaluate the technical frontiers of reconstructing human-like intestinal environments in the lab."

Section 2.3: In this section, it would be helpful to include a practical example to better illustrate the challenges discussed. For instance, the difficulty of studying in vitro host-microbe interactions while simultaneously incorporating adaptive immune systems and 2D or 3D epithelial cultures could be highlighted. In particular, allogeneic reactions caused by MHC incompatibility represent a major limitation when combining immune cells and epithelial models from different donors, and this example would strengthen the discussion.

Response:

We thank the reviewer for the suggestion to include a practical example of the challenges in immune-epithelial integration. We have now incorporated the challenge of allogeneic reactions caused by MHC incompatibility into Section 6 (Paragraph 3). This specific example illustrates why current reductionist models struggle to fully capture adaptive immune complexities and highlights the necessity of moving toward donor-matched or autologous systems to avoid confounding non-physiological immune activation.

We have modified the text as follows: " A major technical hurdle in these immunocompetent systems is the allogeneic reaction caused by major histocompatibility complex (MHC) incompatibility when combining epithelial and immune cells from different donors. These non-physiological immune responses can mask the true regulatory effects of microbial metabolites, representing a significant bottleneck for traditional co-culture platforms."

Section 3.1: It should also be noted that transwell models allow the study of how microbiota-derived metabolites and microorganisms themselves influence epithelial cells and the immune system in the apical and basolateral compartment medium. These systems make it possible to investigate how immune cells conditioned by the microbiome in the basal chamber of the transwell affect epithelial-immune crosstalk. Clarifying these aspects would improve the completeness of the review.

Response:

We thank the reviewer for this insightful comment. We have expanded Section 3.1 to highlight the potential of Transwell models in studying inter-compartmental crosstalk. We now explicitly discuss how these systems allow microorganisms and metabolites to condition basolateral immune cells, which in turn regulate epithelial integrity.

We have revised the text as follows: "The compartmentalized nature of this system makes it possible to investigate how microbiota-derived metabolites or microorganisms applied apically can transition across the barrier to modulate immune cells in the basolateral part of

the system. Such setups allow for the study of complex epithelial-immune crosstalk, where immune cells "conditioned" by the microbial environment feed back onto the epithelium to regulate barrier integrity or inflammatory signaling."

Section 3.3: It would be beneficial to provide more specific examples regarding the possibility of generating apical-out organoids or basal organoid co-cultures. Concrete examples would help readers better understand the practical applications and experimental potential of these approaches.

Response:

In response to this comment we have significantly revised the section on intestinal organoid models (now Section 3.2) to provide a more critical and technically detailed evaluation. We now explicitly contrast the metabolic authenticity of organoids with the metabolic disorder of cancer-derived cell lines, particularly regarding butyrate processing. According to the reviewers' suggestions, we have included concrete examples of advanced organoid configurations, such as microinjection for Phase I junction modeling and apical-out organoids for Phase II pathogen invasion studies. Furthermore, we address the challenge of basolateral access by discussing organoid-derived monolayers and microfluidic chips, highlighting their capacity to integrate autologous immune cells and thus circumvent allogeneic reactions. This revision put the technological discussion in our proposed stepwise framework of barrier failure.

We have revised the text as follows: "Importantly, organoids preserve the oxidative phosphorylation-driven metabolism of a healthy primary epithelium, thus avoiding the Warburg-like glycolytic metabolic disorder found in Caco-2 monolayers…. For instance, the integration of tissue-resident T cells with autologous organoids has recently enabled the study of how the mucosal immune compartment shapes epithelial responses to the microbiota, effectively circumventing the allogeneic reactions typically observed in mismatched donor models."

It would be valuable to include a dedicated section on practical applications of in vitro co-culture systems in biomedical research such as cancer, inflammatory diseases and neurodegenerative diseases. This would help connect the technological developments described in the review with real-world biomedical applications and increase the overall impact of the manuscript.

Response:

We are grateful for this suggestion, as connecting technological developments with real-world applications significantly enhances the impact of our review. As suggested, we have added a new dedicated section, "Section 5: Clinical translation of *in vitro* and ex vivo models". In this section, we discuss how advanced co-culture systems are currently utilized in research on IBD, colorectal cancer, and neurodegenerative diseases. In addition, the drug discovery and precision medicine are included to evaluate the use of PCIS and organoid-derived platforms for high-throughput drug screening and personalized therapy. This addition bridges the gap between model engineering and clinical utility, providing a clearer

perspective on the future directions of the field.

Referee #2 (Remarks for Author):

The topic is timely and the central question is of genuine importance to the field. However, two recent comprehensive reviews (McCoy et al., 2024; Cheng et al., 2025) have already surveyed the landscape of experimental models for intestinal host-microbe interactions in considerable depth. In its current form, this manuscript does not sufficiently differentiate itself from that existing literature. The proposed stepwise framework linking fermentation, in vitro, and ex vivo systems is conceptually reasonable, but it remains largely descriptive. It does not define how biological signals are transformed across model systems, nor does it identify conserved variables that would enable predictive translation between them. Consequently, the manuscript does not resolve the central problem it articulates: the gap between mechanistic insight and human relevance. Substantial revision is required before this work can be considered for publication.
Major comments
1. The title implies a synthesis spanning epithelial biology broadly, yet the analysis is confined to intestinal systems. The authors must either restrict the stated scope explicitly to gut barrier biology throughout the manuscript, or incorporate mechanistic coverage of other epithelial tissues with comparable depth. Current claims of generalizable epithelial regulation are not supported by the evidence presented.

Response:

We acknowledge that the current analysis is focused on the intestinal system, and that the original title and some statements may have suggested a broader applicability to epithelial barriers in general. To avoid overgeneralisation, we have now explicitly limited the scope of the manuscript to the intestinal barrier. At the same time, we would like to emphasise that, beyond a strong focus on barrier function, the review also incorporates a range of experimental models that are not commonly covered in existing literature, which we consider an important addition to the field. As requested, we have changed the title to "Experimental Models for Intestinal Host-Microbe Interactions" to accurately reflect the focus of the review. In addition, we have replaced general terms such as "epithelial cells" with "intestinal epithelial cells" throughout the manuscript to ensure scientific accuracy.

2. The stepwise framework assumes continuity across model systems but does not define it. Fermentation systems quantify metabolite outputs (SCFAs, secondary bile acids, indoles); epithelial models report TEER, junctional proteins, and cytokines; ex vivo systems capture integrated tissue responses. These readouts are not mechanistically aligned, and this creates an unresolved break in causal inference. The authors should introduce conserved mechanistic axes that can be tracked across all three platforms. Appropriate candidates include proteostasis stress (e.g., UPR activation), redox state, and cytokine network topology rather than individual cytokines.

Response:

We sincerely appreciate the reviewer's suggestion to define the mechanistic continuity across the different levels of our framework. We have added three conserved axes: proteostasis and UPR activation, the metabolic redox state, and cytokine network topology as a new dedicated section (now Section 2.3).

By focusing on these axes, we have successfully aligned the biochemical outputs of fermentation with the functional readouts of epithelial and *ex vivo* systems. This approach allows for the consistent tracking of biological signals moving from early intracellular stress to integrated tissue-level responses, ensuring that causal inference is maintained as researchers transition between models of varying complexity.

We have revised the text in section 2.3 as follows: "Although the physical signs of barrier failure are phase-dependent, the underlying biological signals can be tracked across all experimental models through conserved mechanistic axes...This topology enables the identification of consistent signaling across all levels of biological complexity."

3. The manuscript repeatedly frames barrier integrity in terms of reinforcement or disruption. This binary framing obscures the temporal sequence through which barrier failure actually unfolds: an early phase of junctional remodeling and cytoskeletal tension changes; an intermediate phase of mucus layer thinning and increased microbial proximity; and a late phase of immune activation and cytokine-driven permeability amplification. Individual model systems capture discrete phases of this progression, but the manuscript does not map systems onto these transitions. Doing so would substantially strengthen the integrative value of the framework.

Response:

As suggested, we have revised Section 2.1 to define the temporal sequence of barrier failure as a three-phase progression, which includes Phase I (Junctional Remodeling), Phase II (Mucus Depletion and Microbial Invasion), and Phase III (Immune-driven Permeability). By establishing this temporal sequence of framework, we are now able to map individual experimental models onto specific phases of barrier failure throughout the manuscript. This modification significantly strengthens the integrative value of our proposed framework.

We have revised the text in section 2.1 as follows: "The intestinal barrier is not a static gatekeeper but a living assembly that undergoes a complex lifecycle of barrier failure in response to microbial dysbiosis...Recognizing this sequence allows for a more precise mapping of how specific metabolites intervene at different depths of the barrier architecture."

4. While the limitations of in vitro immune modeling are acknowledged, the manuscript does not address how immune activation arises mechanistically from epithelial-metabolite interactions. Receptor-level inputs (GPRs, FXR, AhR) are described in isolation, but their convergence onto shared regulatory nodes is never defined. This represents a significant gap in the mechanistic argument.

Response:

We sincerely thank the reviewer for this profound comment. We agree that describing individual receptor-metabolite pairs in isolation fails to capture the systemic nature of

mucosal immune activation. To address this gap, we have substantially expanded the current Section 2.3 to define the hierarchical signaling network that bridges localized epithelial stress and tissue-level immune responses.

Rather than treating metabolic receptors as independent parts, we now explicitly define how diverse inputs from GPRs, FXR, PXR, and AhR converge onto shared intracellular regulatory hubs. Specifically, we have incorporated the following mechanistic refinements: Firstly, we have clarified how these disparate receptor-level signals intersect at core transcriptional hubs, most notably the NF-κB and STAT3 pathways. Secondly, we have added a detailed discussion on the crosstalk between specific axes, such as how the AhR-NRF2 axis recalibrates the epithelial redox state to regulate the efficacy of GPR-mediated SCFAs. Lastly, we have reorganized Section 2.3 to follow a clear physiological sequence: moving from intracellular sensing (Proteostasis/UPR) to metabolic condition (Redox state), and finally to transcriptional convergence, which dictates the terminal cytokine network topology.

The added paragraph in Section 2.3 is as follows: "The transition from these intracellular signals to systemic immune activation is mediated by the convergence of diverse receptor-level inputs onto the intracellular regulatory hub. Metabolite receptors such as AhR, PXR, and GPR intersect at the core transcriptional level, most notably the NF-κB and STAT3 pathways. For instance, while GPR-mediated SCFAs typically promote barrier integrity, their efficacy is regulated by the activation of the AhR/NRF2 pathway, which recalibrates the epithelial redox state." We believe this addition addresses the reviewer's concern and significantly strengthens the integrative value of our proposed framework.

5. The limitations of individual platforms should be explicitly linked to their impact on gut integrity studies. For gut-on-a-chip systems, constraints including the absence of neuronal and vascular components, variable nutrient absorption, inconsistent mechanical stress profiles, and interspecies differences are noted but their implications for tight junction regulation, paracellular transport, and epithelial resilience are underexplored. Similarly, organoid systems face donor-dependent variability and short culture duration; the effects of these constraints on reproducibility and long-term modeling of barrier dysfunction should be clarified. Listing limitations without connecting them to specific functional consequences limits the utility of this section for readers designing experiments.

Response:

We agree that a critical assessment of model limitations is only valuable if it clarifies the potential impact on readout interpretation. We have thoroughly revised Sections 3.2, 3.3, 3.4, and 4 to explicitly link technical constraints to functional consequences in barrier integrity studies.

Organoid systems (Section 3.2): we now clarify that the short culture duration of organoid-derived monolayers precludes the study of chronic barrier degradation and long-term shifts in paracellular transport. Furthermore, we discuss how donor-dependent variability in baseline TJ expression and mucus thickness can obscure the reinforcement effects of metabolites, hindering the establishment of reproducible dose-response curves.

Anaerobic co-culture systems (Section 3.3): we added a discussion on how the absence of high-speed flow and mechanical strain leads to an underdevelopment of the glycocalyx

and altered TJ maturation, which may result in an overestimation of microbial translocation rates.

Gut-on-a-chip systems (Section 3.4): we have explicitly linked the absence of neuro-vascular feedback to a potential overestimation of metabolite toxicity. Crucially, we explain how inconsistent mechanical stress can physically displace TJ proteins such as ZO-1 and occludin, triggering a barrier failure that is a byproduct of physical shear rather than biological signaling.

Ex Vivo Platforms (Sections 4.1 & 4.2): we have clarified that tissue degradation and apoptosis post-resection can cause paracellular leakage, which is often misinterpreted as a metabolite-induced response. In the Ussing chamber, we now explain how the lack of systemic circulation can trigger oxidative stress that may mask the homeostatic effects of microbial compounds.

By connecting these technical constraints to specific readouts like TJ localization, TEER fluctuations, and paracellular transport, the revised manuscript now provides a practical functional mapping to guide researchers in experimental design and readout interpretation.

6. Current assessments of gut integrity in this review focus on TEER and tight junction proteins. However, mucus thickness, composition, goblet cell differentiation, and epithelial turnover are equally critical determinants of barrier function and are largely overlooked. The manuscript should address whether in vitro and ex vivo models recapitulate mucus dynamics and how these measures complement TEER and protein-based assays to provide a more complete picture of barrier integrity.

Response:

As requested, we have carefully integrated the suggestions regarding the limitations of individual platforms in Sections 3.1, Sections 3.2, Sections 3.3, Sections 3.4, and Sections 4.1, Sections 4.2. Rather than merely listing technical constraints, we have synthesized these insights to explicitly link each model's limitations to its specific functional consequences for gut integrity, such as mucus dynamics, epithelial turnover, and paracellular resilience.

7. Intact tissue platforms such as explants and biopsies preserve native architecture, but systems like precision-cut intestinal slices disrupt the luminal-to-basolateral directionality of microbiota exposure, which compromises biological relevance. Additionally, residual native microbiota is difficult to eliminate in these systems, complicating controlled experimental design. These orientation and microbiome-related pitfalls should be explicitly highlighted when intact tissue models are discussed in the context of barrier function studies.

Response:

We agree that the loss of directionality and the presence of residual microbiota are critical in PCIS models. In the revised Section 4.1, we have synthesized these concerns with overlapping feedback from other reviewer to provide a more robust evaluation. On the one hand, we now explicitly state that the slicing process disrupts the luminal-to-basolateral polarity, potentially leading to non-physiological lateral exposure. We suggest using compartmentalized chambers as a strategic solution to re-establish functional barriers. On

the other hand, we have highlighted the challenge of residual native microbiota and recommended standardized antibiotic depletion to ensure cleaner experimental backgrounds. Therefore, the revised manuscript offers high practical utility for researchers, balancing throughput with physiological accuracy by integrating these into the discussion of PCIS limitations.

8. This is a significant concern. Cancer-derived lines (Caco-2, HT-29, HCT116) exhibit glycolytic bias, altered polarity, and aberrant junctional organization that reshape ligand handling and signaling topology in ways that are not representative of normal intestinal epithelium. Specifically, these systems can exaggerate TGR5-cAMP signaling and uncouple FXR programs from differentiation states. Primary systems, including non-transformed IEC lines and organoids, reveal context-dependent responses that diverge substantially. Across metabolite classes, these differences are mechanistically meaningful and not interchangeable: butyrate is rapidly oxidized in differentiated colonocytes, limiting nuclear HDAC inhibitor accumulation, whereas metabolic rewiring in cancer-derived cells enables this effect; secondary bile acids produce dose-dependent barrier-compatible signaling in normal systems but exaggerated toxicity responses in transformed lines; indole metabolites activate coordinated AhR/PXR programs in primary epithelial systems, whereas cancer-derived lines produce incomplete barrier gene induction or tumor-supportive outputs. Conclusions throughout the manuscript must be stratified accordingly.

Responses:

In response to this comment, we have substantially revised Section 3.1 to incorporate these points. Specifically, we now discuss the 'metabolic rewiring' that leads to unphysiological HDAC inhibition by butyrate in Caco-2 cells. Most importantly, we have included the reviewer's suggestion regarding the aberrant signaling topology in transformed lines, such as the exaggerated TGR5-cAMP signaling and the uncoupling of FXR programs from differentiation states. We have explicitly stated that conclusions from these models must be stratified and validated in primary systems to avoid translational errors.

We have revised the text as follows: "However, conclusions drawn from these systems must be strictly stratified, as their malignant origin imposes significant metabolic and signaling biases that deviate from normal intestinal physiology…Beyond metabolic rewiring, these transformed lines often exhibit an aberrant signaling topology… to ensure they are physiologically representative and to avoid translational misinterpretation."

9. Secondary bile acids are potent TGR5 agonists, whereas FXR is preferentially activated by primary bile acids (e.g., CDCA). The statement that secondary bile acids activate "GPCRs such as TGR5 and FXR" is incorrect and should be revised. Furthermore, secondary bile acid signaling is biphasic: lower concentrations support barrier-compatible signaling, whereas higher concentrations induce membrane and mitochondrial stress, increasing permeability and driving inflammatory signaling. This dose-response transition is entirely absent from the current discussion and represents a mechanistically important omission. Similarly, butyrate is presented as a uniform HDAC inhibitor without accounting for the metabolic state of the cell, which determines whether nuclear accumulation occurs at all. Claims regarding tight junction

transcriptional regulation by butyrate require primary evidence from defined, physiologically relevant systems.

Responses:

We have now fully integrated these mechanistic insights throughout the manuscript to ensure scientific accuracy. On one hand, we have revised the discussion of SBAs to reflect their dose-dependent biphasic effects in Section 2.2. As suggested, we now specify that while lower physiological concentrations of SBAs support homeostasis via the G protein-coupled receptor TGR5, higher levels trigger membrane and mitochondrial stress, leading to increased permeability. We have also corrected the receptor classification, ensuring that FXR is no longer miscategorized as a GPCR and focusing the discussion on the concentration-dependent transition of SBA toxicity. On the other hand, we have significantly expanded Section 3.1 to address the metabolic state-dependency of butyrate signaling. We now contrast the oxidative metabolism of primary colonocytes with the 'Warburg-like' glycolytic state of Caco-2 cells. This distinction clarifies that nuclear accumulation of butyrate as an HDAC inhibitor is often a metabolic artifact of immortalized cells. Consequently, we explicitly state that claims regarding the transcriptional regulation of TJ by butyrate must be validated in primary-derived models to ensure physiological relevance. By distributing these revisions across Sections 2.2 and 3.1, we have ensured that the mechanistic nuances of metabolite-host interactions are grounded in the appropriate biological context.

The text was revised as follows, in Section 2.2: “Notably, the signaling effects of SBAs exhibit a distinct "biphasic" dose-dependent effect that underscores the interdependence of the intestinal barrier…. At higher levels, SBAs can induce membrane and mitochondrial stress, directly increasing paracellular permeability and triggering inflammatory responses.”; In Section 3.1: “For instance, in normal cells, butyrate is rapidly oxidized as a primary energy source…Consequently, assessments are restricted to static TEER and TJ protein assays, which overlook the dynamic barrier function and regeneration of the native intestine.”

10. The manuscript does not distinguish between homeostatic epithelial regulation and disease states such as IBD or colorectal cancer. This distinction is critical, as SCFA, bile acid, and indole signaling diverge substantially across these contexts. Clarification of biological context is required throughout the manuscript, and mechanistic claims should be explicitly qualified by the state in which they apply.

Responses:

We fully agree with the reviewer that the distinction between homeostatic epithelial regulation and disease states is critical. We have systematically revised the manuscript, particularly in Section 5, to ensure all mechanistic claims are qualified by their biological context.

In Section 5.1, we added a discussion on how the pro-inflammatory milieu of IBD can diminish the protective signaling of SCFAs and indoles, contrasting this with their role in a healthy barrier. And then, in Section 5.2, we have expanded the discussion on how the Warburg effect in cancer cells redirects metabolite signaling toward immune evasion rather than barrier reinforcement, highlighting the functional divergence between healthy and

malignant states. In the end, in Section 5.4, we included a concluding statement emphasizing that the functional impact of microbial metabolites is not universal but is dictated by the host's specific inflammatory and metabolic profile. These revisions ensure that the manuscript moves beyond generalized descriptions to provide a nuanced, context-aware framework for barrier function studies.

11. Indole-derived metabolites (IPA, IAld) are presented as uniformly barrier-enhancing. In primary epithelial systems, these metabolites activate AhR (IAld preferentially) and PXR (IPA preferentially), inducing coordinated transcriptional programs that strengthen barrier integrity. In cancer-derived systems, this signaling bifurcates into either incomplete barrier gene induction without restored polarity, or into tumor-supportive outputs. This divergence is mechanistically significant and should be explicitly incorporated into the discussion of indole biology.

Responses:

We thank the reviewer for the insightful comment on the bifurcation of indole signaling. In the revised Section 3.1, we have explicitly addressed how the biological context of the model dictates the functional outcome of indole-derived metabolites such as IPA and IAld. We now clarify that while these metabolites trigger coordinated AhR and PXR transcriptional programs in primary epithelial systems to restore polarity, this signaling is often incomplete or redirected toward tumor-supportive outputs in cancer-derived lines. By contrasting the malignant phenotype of immortalized cells with the physiological behavior of primary-derived models, we provide a mechanistically accurate framework for interpreting microbial-host interaction.

We have revised the text in section 3.1 as follows: "Beyond metabolic biases, these transformed lines often exhibit an aberrant signaling topology. Specifically, cancer-derived systems can exaggerate TGR5/cAMP signaling and uncouple FXR-mediated programs from the cell's differentiation state, leading to distorted responses to SBAs. Similarly, while indole metabolites such as IAld and IPA activate coordinated AhR- and PXR-driven programs to enhance barrier resilience in primary cell lines, this signaling is often incomplete or redirected toward tumor-supportive outputs in immortalized ones. Consequently, the readouts from these models often reflect the behavior of a malignant phenotype rather than a physiological intestine. Mechanistic conclusions regarding microbial metabolites and inter-compartmental crosstalk should therefore be validated in primary-derived models such as organoid-derived monolayers to ensure they are physiologically representative and to avoid translational misinterpretation.

12. Several mechanistic claims rely on reviews or model systems that do not support the conclusions drawn. Specifically: the butyrate-tight junction claims are not supported by the cited review; Reference 11 (ZeXieYin formula study) cannot isolate secondary bile acid effects due to the presence of multiple confounding variables; Reference 14 relies on tumorigenic Caco-2/HT-29 co-culture yet the conclusions are generalized beyond this context. The citation framework should be rebuilt using primary studies with clearly defined and physiologically appropriate model systems.

Responses:

We fully agree with the reviewer that the integrity of the citation framework is important for this manuscript. Consequently, we have systematically rebuilt the evidence base for our mechanistic claims by replacing indirect or confounded citations with more primary studies. Firstly, we have replaced the generalized review citations with primary research articles that utilize defined experimental designs to substantiate the role of butyrate in TJ assembly. Secondly, as suggested, we have removed Reference 11 to eliminate confounding variables and cite primary research utilizing purified SBAs to isolate their specific mechanistic impact on epithelial integrity. In the end, given the structural restructuring of our manuscript, the content previously associated with Reference 14 has been removed. To ensure the highest biological relevance, we have replaced cancer-derived model data with primary studies using normal human intestinal epithelial cells as our references for homeostatic barrier function.

These revisions ensure that our conclusions are drawn in transparent, primary evidence and that the distinction between healthy physiology and malignant signaling is strictly maintained throughout the manuscript.

13. Structural determinants of microbial signaling, including LPS chemotypes and flagellin variants, shape immune activation thresholds in ways that are directly relevant to barrier regulation. Ligands described as "silent" with respect to NF-kB activation are indicated in Figure 1 but are never mechanistically explained. This limits the manuscript's ability to fully integrate microbial sensing with barrier regulation, which is stated as a core aim.

Responses:

We thank the reviewer for pointing out the mechanistic gap between microbial structural determinants and the ‘silent’ signaling depicted in Figure 1. We have reintegrated the discussion of microbial-associated molecular patterns (MAMPs) into Section 2.3, positioning them as a critical regulatory mechanism for the intracellular regulatory hub.

In this revised manuscript, we have explicitly addressed how structural variations in MAMPs, such as LPS acylation patterns, shape the immune activation threshold. We now clarify that while hexa-acylated LPS from pathogens triggers robust NF-κB-mediated inflammation, under-acylated LPS from commensals fails to induce the TLR4/MD2 dimerization necessary for downstream signaling. Rather than a standalone list, these structural determinants are now discussed as part of a conserved mechanistic axis that translates diverse microbial stimuli into specific biological outcomes, thereby fulfilling the manuscript's core aim of integrating microbial sensing with barrier regulation.

The text was revised as follows, in Section 2.3: “Notably, the activation of this hub is also regulated by the structural determinants of microbial ligands like microbial-associated molecular patterns (MAMPs), including LPS, peptidoglycan fragments, and flagellin. Unlike the hexa-acylated LPS from pathogens that trigger robust NF-κB-mediated inflammation, the under-acylated LPS fail to induce TLR4/MD2 dimerization, which decouples microbial sensing from inflammatory signaling, shifting it toward immune tolerance.”

14. GPCR and nuclear receptor signaling are discussed throughout without defining receptor

distribution across intestinal epithelial and immune cell compartments. Because receptor expression determines signaling outcome, this information is essential to the mechanistic arguments being made. The authors should provide a structured mapping of receptor expression across relevant cell types, ideally as a tabulated summary, to anchor the signaling claims made throughout the manuscript.

Responses:

We agree that defining receptor distribution is essential for anchoring the mechanistic claims made throughout the manuscript. We have now added a table that maps the expression of key GPCRs and nuclear receptors across both epithelial and immune cell compartments. This table explicitly links receptor location to specific signaling outcomes and barrier phases. In addition, in Section 2.2, we have revised the introductory paragraph of Section 2.2 to emphasize that this spatial partitioning dictates the functional impact of metabolites. This structured mapping now serves as a foundational reference for the disease-specific and model-specific discussions in the subsequent sections, ensuring all claims are physiologically grounded.

Minor comments

The authors should ensure figure legends are consistent with the mechanistic content of the main text, particularly Figure 1, where structural ligand categories are depicted but not explained in the accompanying discussion.

Responses:

We have revised the figure legend of Figure 1 to ensure full consistency with the newly added mechanistic discussion in the main text (Section 2.3). Specifically, we have added a concise explanation of the structural categories of microbial ligands, clarifying how structural variations such as LPS acylation patterns determine the threshold for NF-κB activation versus immunological tolerance. This ensures that the visual representation of ‘silent’ ligands is now explicitly supported by both the figure legend and the accompanying discussion.

23rd Apr 2026

Dear Dr. Li,

Thank you for the submission of your manuscript to EMBO Molecular Medicine. I am pleased to inform you that we will be able to accept your manuscript pending the following final amendments:

1) Authorship: The last author, Paul de Vos, should be designated as a co-corresponding author.
2) Figures:
- Please remove all figures from the manuscript and leave only their legends at the end of the file.
- We have sent the figures for the final adjustments to our graphic designer, who will contact you for your approval.
3) Tables: Please place Tables after figure legends.
4) Information about funding, if relevant, should be added to "Acknowledgments" and in our submission system.
5) Remove "Declaration of generative AI and AI-assisted technologies in the writing process" and place the current text in "Acknowledgements".
6) Correct the reference citation in the text and reference list. In the text, a reference should be cited by author and year of publication. Include a space between a word and the opening parenthesis of the reference that follows. In the reference list, citations should be listed in alphabetical order. Where there are more than 10 authors on a paper, 10 will be listed, followed by "et al.". Please check "Author Guidelines" for more information.
https://www.embopress.org/page/journal/17574684/authorguide#referencesformat
7) As part of the EMBO Publications transparent editorial process EMBO Molecular Medicine will publish online a Review Process File (RPF) to accompany accepted manuscripts. This file will be published in conjunction with your paper and will include your point-by-point response and all pertinent correspondence relating to the manuscript. Let us know whether you agree with the publication of the RPF.
8) Please provide a point-by-point response letter to my comments (as Word file).

I look forward to receiving the revised version of your manuscript.

Yours sincerely,

Zeljko Durdevic

Zeljko Durdevic
Senior Editor
EMBO Molecular Medicine

*** IMPORTANT INFORMATION ***

When submitting your revised manuscript, please include:

1) a .doc formatted version of the manuscript text (including Figure legends and tables)
2) Separate figure files
3) a letter INCLUDING the reviewer's reports and your detailed responses to their comments.

Also, and to save some time should your paper be accepted, please read below for additional information regarding some features of our research articles:

1) Glossary: EMBO Molecular Medicine articles will be accompanied by a glossary explaining some of the terms used for laymen. I identified the following:

____, ____, ____

Could you please help us in identifying terms that may need an "explanation" other terms that we can add to the glossary.

2) For more information: This is a short list of related web links for further consultation by the readers. Could you identify some relevant ones? Examples are patient associations, OMIM related links, databases, authors websites, etc.

3) Pending issues: At the end of each article we will have a box highlighting issues that still need further studies and where research efforts should converge (we call this the Pending issues box). From my reading I would say:

----

but I can see there may be many more. Could you work on this as well?

4) Disclosure and competing interest statement: Please include a statement declaring any competing commercial interests in relation to your submitted work.

5) Please note that we now mandate that all corresponding authors list an ORCID digital identifier. This takes <90 seconds to complete. We encourage all authors to supply an ORCID identifier, which will be linked to their name for unambiguous name identification.

Currently, our records indicate that the ORCID for your account is 0000-0003-0236-8581.

Please click the link below to modify this ORCID:
Link Not Available
-

Thank you,

Zeljko Durdevic

Zeljko Durdevic
Editor
EMBO Molecular Medicine

***** Reviewer's comments *****

Referee #1 (Remarks for Author):

I have no further comments for the authors

Referee #2 (Remarks for Author):

All the concerns raised have been addressed substantively.
The revision is thorough, and significantly improves the manuscript's mechanistic depth, clinical relevance, and critical evaluation of model limitations. The authors have restructured key sections, added new conceptual frameworks (three-phase barrier failure, conserved mechanistic axes), and corrected factual errors (bile acid receptors, butyrate metabolism).
I am supportive of its publication

Responses to the editor

1) Authorship: The last author, Paul de Vos, should be designated as a co-corresponding author.

Responses: As requested, Paul de Vos has been designated as a co-corresponding author. The title page and the submission system have been updated accordingly to include his contact information.

2) Figures:

- Please remove all figures from the manuscript and leave only their legends at the end of the file.
- We have sent the figures for the final adjustments to our graphic designer, who will contact you for your approval.

Responses: As requested, all figures have been removed from the manuscript, and only their legends are at the end of the manuscript. Many thanks for letting us know about the upcoming communication with the graphic designer.

3) Tables: Please place Tables after figure legends.

Responses: As requested, all Tables have been placed after figure legends in the manuscript.

4) Information about funding, if relevant, should be added to "Acknowledgments" and in our submission system.

Responses: As this is a review article and was not supported by specific external research grants, we have added a statement to the "Acknowledgments" section and updated the submission system to indicate that no specific funding was received for this work.

5) Remove "Declaration of generative AI and AI-assisted technologies in the writing process" and place the current text in "Acknowledgements".

Responses: As requested, we have removed "Declaration of generative AI and AI-assisted technologies in the writing process" and placed the current text in "Acknowledgements".

6) Correct the reference citation in the text and reference list. In the text, a reference should be cited by author and year of publication. Include a space between a word and the opening parenthesis of the reference that follows. In the reference list, citations should be listed in alphabetical order. Where there are more than 10 authors on a paper, 10 will be listed, followed by "et al.". Please check "Author Guidelines" for more information.
https://www.embopress.org/page/journal/17574684/authorguide#referencesformat

Responses: Thank you for your suggestion. As requested, we have thoroughly revised the reference list and in-text citations to strictly adhere to the EMBO Molecular Medicine guidelines.

7) As part of the EMBO Publications transparent editorial process EMBO Molecular Medicine will publish online a Review Process File (RPF) to accompany accepted manuscripts. This file will be published in conjunction with your paper and will include your point-by-point response and all pertinent correspondence relating to the manuscript. Let us know whether you agree with the publication of the RPF.

Response: I confirm that I agree with the publication of the Review Process File (RPF) alongside my manuscript.

8) Please provide a point-by-point response letter to my comments (as Word file).

27th Apr 2026

Dear Dr. Li,

We are pleased to inform you that your manuscript is accepted for publication and is now being sent to our publisher to be included in the next available issue of EMBO Molecular Medicine.

Your manuscript will be processed for publication by EMBO Press. It will be copy edited and you will receive page proofs prior to publication. Please note that you will be contacted by Springer Nature Author Services to complete licensing information.

Should you be planning a Press Release on your article, please get in contact with embo_production@springernature.com as early as possible in order to coordinate publication and release dates.

If you have any questions, please do not hesitate to contact the Editorial Office. Thank you for your contribution to EMBO Molecular Medicine.

Yours sincerely,
Zeljko Durdevic

Zeljko Durdevic
Senior Editor
EMBO Molecular Medicine
